# Modeling and maximizing influence diffusion in social networks for viral marketing

**DOI:** 10.1007/s41109-018-0062-7

**Published:** 2018-04-10

**Authors:** Wenjun Wang, W. Nick Street

**Affiliations:** Department of Management Sciences, University of Iowa, 21 E Market St, Iowa City, IA 52242 USA

**Keywords:** Influence diffusion, Influence maximization, Social network, Viral marketing

## Abstract

Modeling influence diffusion in social networks is an important challenge. We investigate influence-diffusion modeling and maximization in the setting of viral marketing, in which a node’s influence is measured by the number of nodes it can activate to adopt a new technology or purchase a new product. One of the fundamental problems in viral marketing is to find a small set of initial adopters who can trigger the most further adoptions through word-of-mouth-based influence propagation in the network. We propose a novel *multiple-path asynchronous threshold* (MAT) model, in which we quantify influence and track its diffusion and aggregation. Our MAT model captures not only direct influence from neighboring influencers but also indirect influence passed along by messengers. Moreover, our MAT framework models influence attenuation along diffusion paths, temporal influence decay, and individual diffusion dynamics. Our work is an important step toward a more realistic diffusion model. Further, we develop an effective and efficient heuristic to tackle the influence-maximization problem. Our experiments on four real-life networks demonstrate its excellent performance in terms of both influence spread and time efficiency. Our work provides preliminary but significant insights and implications for diffusion research and marketing practice.

## Introduction

People live in various social networks, and share information and ideas with friends in the form of word-of-mouth (WOM) communication. New technologies and various social media rapidly penetrate into every aspect of our daily life, and provide us new channels and great convenience to exchange information and express opinions. They disseminate massive volumes of information over different social media, and spread influence to each other. As social media becomes prevalent, its influence on business, politics and society becomes evident and significant. How new innovations, behaviors, and diseases spread through social networks has a long history of study in social sciences. Research in this area has exploded and drawn considerable attention from many disciplines over the last decade. Many models of information and influence diffusion have been proposed for a wide variety of applications, such as viral marketing (Kempe et al. 2003; Bhagat et al. 2012), cascading behavior and prediction (Leskovec et al. 2007; Cheng et al. 2014), information spreading (Morales et al. 2014), outbreak detection (Leskovec et al. 2007), etc. In this paper, we look into modeling influence diffusion in the setting of viral marketing in business.

In essence, viral marketing is a process of influence diffusion over social networks. An effective viral marketing campaign requires that marketers identify individuals with high social networking potential. A general setting can be depicted as follows: a company would like to market a new product, in the hopes that it would be adopted by as many people as possible in a target social network. The company chooses a small number of influential individuals as the initial adopters/seeds by giving them free/discounted samples of the product and encourages them to recommend the product to their friends, hoping that their friends would be influenced to purchase the product, then influence their friends, and so on. As a result, the influence would propagate through the network and trigger widespread adoption in the end of the diffusion process. Viral marketing is termed to describe such a marketing technique that induces the users in a social network to pass on a marketing message (viral ad) to others so as to achieve the largest influence spread in terms of product sales or brand awareness.

Viral marketing is driven by WOM communication and enhanced by network effects. Survey-based statistical research has shown very strong support for the hypothesis that network linkage can directly affect product/service adoption (Hill et al. 2006; Iyengar et al. 2011). The crucial factor to the success of viral marketing is for the marketers to identify the most influential set of initial adopters. Researchers investigated different approaches to seeding campaigns for marketing practice (Hill et al. 2006; Iyengar et al. 2011; Libai et al. 2013; Rand and Rust 2011; Peres 2014). These studies significantly enhance our understanding of WOM behavior and effects on promoting viral marketing. However, the approach these researchers commonly use or rely on is explanatory modeling. They apply various field experiments and/or statistical models to data for testing some causal hypotheses or correlations between variables. For example, researchers find support for the hypothesis that referrals from strong ties are more influential in receivers’ decision-making than those from weak ties (Brown and Reinegen 1987), and that high-degree seeding remains the most successful strategy (Hinz et al. 2011). Nevertheless, can we create a robust seeding strategy that outperforms the high-degree seeding? Is it possible to find the optimal or suboptimal seeding strategy? Can we estimate the cascade of adoption given any set of initial adopters? Most of these qualitative approaches and empirical studies fail to give answers to these important questions in an operational manner due to the lack of an explicit/realistic influence-diffusion model.

While most marketing researchers focus on investigating WOM diffusion processes using various explanatory models and/or qualitative methods, researchers from computer science and other related fields aim to explicitly build influence-diffusion models so as to maximize and/or predict influence spread. Influence maximization as an important algorithmic technique for viral marketing was first posed by Domingos and Richardson (2001, 2002), in which they applied Markov random fields to model the influence among customers and then choose the best marketing plan to maximize the profit. In their seminal paper (Kempe et al. 2003), Kempe et al. formulate it as a discrete optimization problem: given a social network, a stochastic diffusion model, and a number *K* (also called budget), the objective is to find the seed set of *K* initial adopters (of a new product) who can trigger the largest cascade of further adoptions, in which the influence diffusion process unfolds in discrete time steps as described by the diffusion model.

This influence-maximization (IM) problem has attracted a great deal of attention and extensive studies. To address this problem, two key components are indispensable. First, we need to build a realistic influence-diffusion model that captures in detail the diffusion process and the activation mechanism of WOM marketing. Second, we need to develop an effective and efficient algorithm that enables us to find the optimal or suboptimal seed set under the diffusion model. Kempe et al. (2003) approach the problem under two widely-studied stochastic diffusion models: *independent cascade* (IC) model and *linear threshold* (LT) model. They show that this optimization problem is NP-hard for both the IC and LT models, and provide the first provable approximation guarantees for heuristic algorithms. Their work laid theoretical and algorithmic foundations for understanding influence diffusion and addressing the IM problem. Since then, there has been a large amount of follow-up work in two main directions: (1) extensive research has been done to study various extensions to the IC and LT models (Bhagat et al. 2012; Goyal et al. 2010; Chen et al. 2011; Borodin et al. 2010; He et al. 2012; Gayraud et al. 2015); (2) a large number of algorithms have been proposed to improve efficiency in finding the *K*-seed set (Leskovec et al. 2007; Chen et al. 2010; Goyal et al. 2011b; Jiang et al. 2011; Wang et al. 2012). These extensions are the substantial complement of the classic IC and LT models. However, there exist some significant limitations in these models.

First, they approach a social entity’s adoption likelihood from a confined scope, considering only the direct influence from the activated neighbors of the entity. However, a social entity’s adoption decision could be influenced by other adopters who are not social neighbors (Fang et al. 2013), e.g. through *structural equivalence* (Burt 1987) and *three-degrees-of-influence* (Christakis and Fowler 2007). In reality, as described in the *network coproduction model* (Kozinets et al. 2010), *WOM messages do not flow unidirectionally from seeded consumers to others, but rather may be exchanged among any connected consumers in the network*. In other words, a node in the network could be influenced and activated by inactive neighbors who pass on the influence from other influencers. The IC and LT models fail to capture such *indirect influence* passed along by messengers who may or may not be activated. The findings of Fang et al. (2013) suggest that diffusion models relying exclusively on direct influence are limited in predictive power. We argue that messengers play an important role in influence diffusion for viral marketing, which should be taken into account to build a more realistic influence-diffusion model.

Second, both IC and LT models have an undesirable time-invariant property (Chen et al. 2013). Delaying the activation attempts of any node in the IC model and delaying adding the influence weights of some out-links of a newly activated node in the LT model would not change the distribution of the final active set. This time-invariant property is not realistic. Goyal et al. (2010) measure the number of actions that propagate between pairs of neighbors in Flickr at different time intervals. They find that there is an exponential decay in the number of actions propagated as the time elapses. In practice, the time constraint and spreading speed are always critical concerns of marketers since they are closely related to profit and competition. In fact, influence not only decays with time but also attenuates along diffusion paths. As indicated in the *three-degrees-of-influence* phenomenon (Christakis and Fowler 2007), while it keeps propagating up to a social horizon of three hops, peer influence dissipates along the diffusion path. Supportive experimental studies have continued to appear (Morales et al. 2014; Bond et al. 2012; Liu et al. 2012).

Third, the IC and LT models (and most generalizations) fail to capture the individual temporal diffusion dynamics. In the IC model, each newly activated node gets a single chance to simultaneously execute an activation attempt to each of its inactive out-neighbors in one time step. In the LT model, an inactive node checks each of its in-neighbors at the same time to compute the total influence weight in any time step. Both of them are synchronous diffusion models. In fact, WOM communication generally takes place in an asynchronous way. Some people are much more active in WOM messaging than others, and people communicate much more frequently with their family members and close friends than regular friends. Even if a person sends out a message simultaneously to all her friends on Twitter, they may log in and check out the message at different times. As shown by Iribarren and Moro in their viral email experiment (Iribarren and Moro 2009), there is large heterogeneity in individual human response time which has great impact on the dynamics of collective information diffusion.

There is another subtle but more fundamental issue in existing models. WOM’s effectiveness as an information source for consumers can be broken down into WOM’s reach and impact. Unlike epidemic spreading in which each exposure acts independently, WOM’s impact is usually derived from *social reinforcement*, where repeated exposures continue to have large marginal effects on adoption (Centola 2010; Romero et al. 2011). We thus consider product adoption as a three-stage process of influence accumulation. The first stage is *awareness*, where an inactive entity gets exposed to the WOM message. The second stage is *aggregation*, in which the entity gets reinforced with more and more WOM exposures. The last stage is *activation*, where the entity adopts the product when the accumulated influence is greater than a certain threshold of the entity. We refer to this procedure as the *3A process*, and attempt to explicitly reveal this process in an incrementally aggregate manner.

We aim at developing a more realistic influence-diffusion model to address these issues. We arrive at a *multiple-path asynchronous threshold* (MAT) model. In this model, we naturally integrate the three stages of the *3A process*, and quantify in an incremental manner the aggregate consequences from informational influence to activation on the basis of complex WOM communication. We consider both direct and indirect influence, take into account influence attenuation along diffusion paths and influence decay with time, and model the individual temporal diffusion dynamics using a contact-frequency-based Poisson process. Further, we develop a novel approximation algorithm to address the influence-maximization problem under our MAT model, and conduct extensive experiments on four real-life networks. Our work provides preliminary but important insights and implications for viral marketing and diffusion research in other fields.

## Related research

Viral marketing is an important and cost-effective marketing technique in business. There are a large number of studies in the literature addressing influence diffusion and viral marketing. Detailed surveys can be found in (Chen et al. 2013; Kempe et al. 2015; AlSumaidan and Ykhlef 2016). Here we focus on several representative models and algorithms most relevant to our work.

### Diffusion models

Kempe et al. published a seminal paper on maximizing influence spread in social networks (Kempe et al. 2003). They formulate influence maximization as a discrete optimization problem, and investigate the step-by-step dynamics of adoption under two basic diffusion models: the *independent cascade* (IC) model and the *linear threshold* (LT) model.

The IC model takes the social network *G*=(*V*,*E*), the influence probability *p*(·) on each link, and the initial seed set *S*_0_ as input, and the influence-diffusion process unfolds in discrete time steps according to the following randomized propagation rule. At each time step *t*_*i*_ (*i*≥0), each newly-activated node *u* is given a single chance to activate each currently inactive out-neighbor *v* independently. It succeeds with the pre-specified influence probability *p*_*uv*_. If successful, then *v* becomes active in step *t*_*i*+1_. But whether or not *u* succeeds, it cannot make any further attempts to activate *v* in subsequent steps. The diffusion process runs until no new nodes are activated. Such diffusion behavior is referred to as *simple contagion* in social science, in the sense that activation could be triggered by a single influencer independently. This model is suited for epidemic spread since an individual may become infected once exposed to the virus. For viral marketing, it does not fit very well. With respect to the *3A process* described previously, the IC model reflects the brand-awareness stage, but fails to explicitly capture the aggregation or activation stages since most people cannot be convinced to adopt a new product after a single WOM interaction.

In the LT model, every link (*u*→*v*) of the social network *G* is associated with an influence weight *b*_*uv*_∈ [0,1], indicating the influence significance of *u* on *v*. Before the diffusion process starts, each node *v* is assigned a threshold *θ*_*v*_ uniformly at random in the range [ 0,1]. The threshold can be interpreted as the personal latent tendency of a node to adopt a new product. Once the initial seed set *S*_0_ is given, the diffusion process unfolds deterministically in discrete time steps. In any step, an inactive node *v* becomes active if and only if the total influence weight of its active in-neighbors $N_{v}^{in}$ is greater than or equal to its threshold *θ*_*v*_, i.e., $\sum _{u\in N_{v}^{in}} b_{uv}\geq \theta _{v}.$ The diffusion process continues until no further activation is possible. As opposed to the IC model, the LT model captures *complex contagion*, in which an individual is usually not activated until she receives positive reinforcement from multiple influencers. The LT model is more suitable for viral marketing than the IC model in this regard.

These two classic models lie at the core of a large number of generalizations in the subsequent work. Bhagat et al. (2012) adapt the LT model to what they called *linear threshold with color* (LT-C) model. They show that there exist *tattlers* who are activated without adopting the product themselves. These tattlers serve as information bridges in influence propagation and significantly affect product adoption. It is no doubt that distinguishing tattlers from adopters is an important step toward a more realistic model. However, a tattler does not have to be activated in order to spread influence in reality. For example, Tom bought an iPhone, and told his friend Jeff that it is cool and he likes it. When Jeff chats with their friend Nick at lunch, he simply tells Nick that Tom just bought an iPhone and he really likes it. Jeff does not have to be activated or form his opinion about the iPhone, but he does pass Tom’s influence on to Nick indirectly. Just like Jeff, many tattlers are simply *messengers* who pass WOM messages on to their friends and implicitly pass *indirect/passive/informational* influence around. This is an important feature of WOM communication, as described in the *network coproduction model* (Kozinets et al. 2010). In fact, messengers play an important role in WOM marketing, and should be distinguished and included explicitly in influence-diffusion modeling.

To incorporate the temporal aspects observed in diffusion dynamics, Chen et al. (2012) investigate time-critical influence maximization. They associate a degree-based or randomly-selected meeting probability to each edge to reflect the time-delayed diffusion dynamics. It is a realistic step to incorporate temporal diffusion dynamics of individual nodes. However, this model has limitations on the following two respects. First, the meeting probability would be meaningless if there is no deadline constraint or the deadline constraint is set to a relatively large number. As noted in previous section, the IC model has a time-invariant property, i.e., delaying the activation attempts of any nodes would not change the distribution of the final active set. In other words, the meeting probability has an effect in their model only because of the deadline constraint, which apparently underestimate the impact of individual temporal dynamics on influence diffusion. We argue that it would make more sense to associate the temporal diffusion dynamics with the temporal influence decay. Second, they fail to capture the *contact frequency* in their meeting probability. For most weighted social networks, the weight on a link is a natural and important indicator that describes the strength of the relationship and/or the contact frequency. A realistic diffusion model should incorporate the weight information to better capture the individual temporal diffusion dynamics.

There are many other extensions of the classic IC and LT models to different scenarios. Chen et al. (2011) extend the IC model to incorporate the emergence and propagation of negative opinions. Chen et al. (2015) adapt the IC model to the topic-aware influence propagation, in which the influence probability on each link is determined by the respective topic distribution. The LT model has been extended to address the influence-maximization problem under competition (Borodin et al. 2010; He et al. 2012). Gayraud et al. (2015) extend both the IC and LT models to study influence diffusion in dynamic networks. However, all the aforementioned models are probabilistic models. None of them explicitly quantify the influence or directly compute the aggregation of influence to reveal the 3A process from *awareness* →*aggregation* →*activation*.

### Influence maximization

The IM problem is formally described as follows: Given a social network *G*=(*V*,*E*) with a total of *n* nodes (i.e., |*V*|=*n*), a stochastic diffusion model on *G*, and a number *K*(*K*≪*n*) that specifies the number of seed nodes (e.g., initial adopters of a new product), find the seed set of *K* nodes to be activated first so that they can trigger the largest cascade of further adoptions in the network. More precisely, let *S*_0_ denote a seed set, *ϕ*(*S*_0_) denote the final active set generated by the stochastic diffusion model, and *σ*(*S*_0_)=*E*(|*ϕ*(*S*_0_)|) denote the *influence spread* of seed set *S*_0_, which is the expected size of the final active sets of all random runs of influence diffusion under the given diffusion model. Then the IM problem is to find the optimal seed set *S*^∗^ such that

1 $$ S^{*}=\underset{{S_{0}\subseteq V,\; |S_{0}|=K}}{\arg\max}\sigma (S_{0}).  $$

The IM problem is NP-hard for both the IC and LT models (Kempe et al. 2003). Moreover, the influence spread function *σ*(·) in both the IC and LT models has two important properties: *monotonicity* and *submodularity*. A set function *f*:2^*V*^→*R* is monotone if for any subsets *S*⊆*T*⊆*V*, *f*(*S*)≤*f*(*T*), which in this context means that adding more nodes to a seed set cannot reduce the size of the final activated set. The set function *f* is submodular if for any subsets *S*⊆*T*⊆*V* and any element *u*∈*V*∖*T*, *f*(*S*∪{*u*})−*f*(*S*)≥*f*(*T*∪{*u*})−*f*(*T*). Submodularity can be understood as diminishing marginal return, which in this context means the marginal gain of node *u* when added to a seed set *T* cannot exceed the marginal gain when adding it to a subset *S*⊆*T*. It is shown that the influence spread function *σ*(·) for any general cascade model or general threshold model is always monotone, but the general models may not satisfy the submodularity property (Chen et al. 2013). As an example, consider the *fixed threshold model* on a simple graph that contains only three nodes {*u*,*v*,*i*} and two links {(*u*→*i*), (*v*→*i*)} with influence weight *b*_*ui*_=*b*_*vi*_=0.5. In a fixed threshold model, each node has a fixed threshold instead of a threshold uniformly selected at random for each run of influence diffusion. For this example, node *i* has a fixed threshold *θ*_*i*_=0.8. Then we can calculate the influence spread and obtain: *σ*(*∅*)=0, *σ*({*u*})=*σ*({*v*})=1 (since *b*_*ui*_=*b*_*vi*_=0.5<0.8=*θ*_*i*_, node *i* is not activated), and *σ*({*u*,*v*})=3 (since *b*_*ui*_+*b*_*vi*_=1>0.8=*θ*_*i*_, node *i* is activated). Now let us set *S*=*∅* and *T*={*u*}. Then we have *σ*(*S*∪{*v*})−*σ*(*S*)=1<2=*σ*(*T*∪{*v*})−*σ*(*T*). It indicates the marginal gain of node *v* when added to *T* exceeds the marginal gain when adding it to *S* (*S*⊆*T*). Therefore, the influence spread function *σ*(·) for this fixed threshold model is not submodular.

After exploiting these properties, Kempe et al. present a simple greedy hill-climbing algorithm to find the *“optimal”K*-node seed set. As it relies on Monte Carlo (MC) simulation to compute the influence spread, we refer to it as MC-Greedy hereafter. As shown in Algorithm 1, MC-Greedy starts with an empty seed set (Line 2), and runs *K* rounds to generate a set of *K* seed nodes. In each round, it sweeps over each node *u*∈*V*∖*S* to compute the influence spread *σ*(*S*∪{*u*}). Since the exact computation of influence spread under the IC and LT models is *♯P*-hard (Chen et al. 2010), they run MC simulation *R* times to estimate the influence spread (Line 6-10). Finally, the node that together with current seed set *S* generates the maximum influence spread is added to *S* (Line 12).


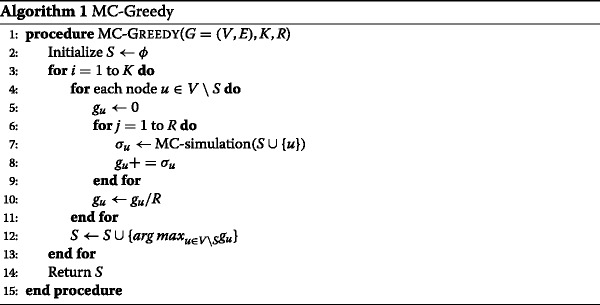


For any model (such as the IC and LT models) in which the influence spread function *σ*(·) is monotone and submodular, MC-Greedy approximates the optimal within a factor of (1−1/*e*−*ε*) for any *ε*>0. This is a performance guarantee at least 63% of optimal. However, this algorithm suffers from a very high computational cost. It requires *O*(*nK*) evaluations of influence spread, and each evaluation needs to run *R* simulations of the diffusion process. Not only is each simulation generally time-consuming, but *R* also has to be large enough to maintain the effectiveness of the algorithm. This performance inefficiency makes it infeasible even for medium-sized networks of tens of thousands of nodes and edges.

To improve its efficiency, one strategy is to reduce the number of influence-spread evaluations. For example, Leskovec et al. (2007) exploit submodularity and present a *Cost-Effective Lazy Forward* (CELF) scheme that significantly reduces the number of evaluations. Unfortunately, CELF and its extensions CELF++ (Goyal et al. 2011a) are still not scalable due to the overhead of Monte Carlo simulation. Therefore new heuristic algorithms that avoid the simulation are needed. This strategy requires exploiting specific aspects of the diffusion model and the network structure. Wang et al. (2012) propose a *maximum influence arborescence* (MIA) algorithm for the IC model. The main idea is to use local arborescence structures of each node to approximate the influence propagation. Chen et al. (2010) present a *local directed acyclic graph* (LDAG) algorithm for the LT model, in which they construct a LDAG for each node and restrict influence diffusion to a node only through its LDAG. Goyal et al. (2011b) present a more efficient heuristic called SIMPATH for the LT model. This algorithm is built upon the CELF optimization.

## Methodology

In our previous work (Wang and Street 2014; 2015a), we develop a *reachability-based* influence-diffusion model to exploit the implicit knowledge of influence-based connectivity and vertex-pair similarity encoded in the network graph topology. This model has been successfully used to uncover influence centrality and community structure in social networks (Wang and Street 2015a, b). In this *reachability-based* model, a node’s influence significance is measured by the total amount of *information* the node spreads over its neighborhood within a pre-specified depth limit. Roughly speaking, it focuses on information’s visibility (e.g., brand awareness) regardless of information’s effect (e.g., adoption). In this paper, we investigate *activation-based* influence-diffusion modeling for viral marketing, in which a node’s influence significance is measured by the total number of nodes it can activate to adopt a new technology or purchase a new product. We adapt our *reachability-based* influence-diffusion model to the viral-marketing scenario and propose a novel, more realistic *activation-based* influence-diffusion model. We call it the *multiple-path asynchronous threshold* (MAT) model, in which we quantify influence and track its diffusion and aggregation. We integrate direct and indirect influence, and instantiate the 3A process to model complex WOM communication and its effects. Moreover, we naturally incorporate in our MAT model the influence attenuation along diffusion paths, influence decay with time, and individual temporal diffusion dynamics based on the relationship strength or contact frequency between a node of interest and its neighbors. Further, we develop an effective and efficient heuristic, called *IV-Greedy*, to address the influence-maximization problem.

### MAT Model

Our model is applicable to both undirected/directed and unweighted/weighted networks. Without loss of generality, let *G*=(*V*,*E*,*W*) be a directed and weighted network with |*V*|=*n* and |*E*|=*m*. The weight *w*_*uv*_∈*W* associated with a directed edge (*u*→*v*) represents the relationship strength of node *u* over node *v*, e.g., the frequency that *u* calls or emails *v*. The relationship strength is usually asymmetric in directed networks. For any unweighted network, we regard it as a weighted network with a weight of 1 on each link. For any undirected edge between two nodes, we replace it with a pair of directed links pointing to each other, associated with the same weight as the original weight on the undirected edge. The influence is propagated through *out-links*.

We categorize all the nodes in the network into two types: influencers and messengers. An *influencer* is an active node (product adopter) that can originate and spread its influence in the network. A *messenger* is an inactive node that may acquire influence and also pass on to her friends the influence it receives from influencers or other messengers. Once a messenger acquires influence that is greater than or equal to its threshold, it is activated and turns into an influencer who starts to spread out its own influence to others. It is noted that an influencer can not only diffuse its own influence to others but also act as a messenger passing along the influence from other influencers. Our model captures an important characteristic of WOM communication (Kozinets et al. 2010) in that: *anyone (either active or inactive) can pass along WOM messages and potentially influence the recipient*. In other words, a node can be activated by not only *direct influence* from its active neighbors (influencers) but also *indirect influence* passed along by its inactive neighbors (messengers). This is a distinguishing, more realistic feature built in our MAT model.

From a graph-theoretic point of view, the influence diffusion from each influencer can be regarded as a batch of random walks around the neighborhood of the influencer. Each walk explores one diffusion path from the influencer and passes on the influence to the nodes it visits along the path. All walks together constitute an *influence-diffusion tree* with the influencer as the root node. To model the complex WOM communication in a more realistic manner, we incorporate in the diffusion tree/process of each influencer several important rules/mechanisms as follows:

- *No cycles or loops are allowed*. It makes sense since no one should repeatedly send out the same WOM message to her friends when the message she sent out circulates back to herself;
- *A node may be visited multiple times along different diffusion paths*. It resembles that the message/influence may be passed on to the same person via different chains of friends. This is a more realistic imitation of WOM messaging and social reinforcement in terms of repeated exposures (Romero et al. 2011);
- *The diffusion tree of one influencer may contain other influencers*. That is because influencers also act as messengers passing along the message/influence from other influencers;
- *Influence dissipates along diffusion paths*. Moreover, the diffusion process stops after three hops from the influencer to match the well-known *three-degrees-of-influence* phenomenon (Christakis and Fowler 2007);
- *Influence diffusion from a parent node to its child nodes is asynchronous*. This is an important mechanism implemented in our model to capture the individual diffusion dynamics.

It is worth pointing out that: 1) the influence-diffusion processes of different influencers are independent of each other; 2) influence not only attenuates along diffusion paths but also decays over time. We elaborate below how we quantify influence and keep track of its diffusion and aggregation following the rules described above.

The relationship strength, as indicated by the weight on edges, may vary significantly among family members, close friends, casual acquaintances, and so on. Stronger relationship implies stronger influence potential in general. To quantify the relationship strength on influence, we define a *normalized influence weight* that measures the fraction of influence a node receives from a specific in-neighbor relative to the total influence it may receive from all of its in-neighbors. Given a directed edge *u*→*v* with a raw weight *w*_*uv*_, and letting $N_{v}^{in}$ denote the set of node *v*’s in-neighbors, the normalized influence weight $\hat {w}_{uv}$ is defined as

2 $$ \hat{w}_{uv}=\frac{w_{uv}}{\sum_{k\in N_{v}^{in}} w_{kv}}.  $$

To quantify the influence attenuation along a diffusion path, we define a *depth-associated attenuation coefficient*

3 $$ \alpha = d^{-2},  $$

where *d* is the depth (number of hops) from an influencer to the node of interest along a diffusion path. It is the same as what we define in the *reachability-based* influence-diffusion model (Wang and Street 2014; 2015a). It can be interpreted as the probability of an influencer’s influence reaching a node *d* hops away, as analogous to the probability of a center node linking to a node at a fixed distance *d* of its concentric scales of resolution, which is proportional to *d*^−2^ as depicted in (Easley and Kleinberg 2010). From a probabilistic perspective, letting a random variable *X*_*i*_ denote the total amount of influence an influencer *i* spreads, and letting a random variable *Y* denote any diffusion path from *i*, then we can for now quantify influencer *i*’s total influence as the conditional expectation

4 $$ E[\!X_{i}]=\sum\limits_{y} E[\!X_{i}|Y=y]P\{Y=y\},  $$

where *y* is a diffusion path from influencer *i* to a destination node *j*. *E*[ *X*_*i*_|*Y*=*y*] is the expected influence *j* acquires along the path *y*, which is estimated by the chain product of the normalized influence weights of the corresponding links that constitute the diffusion path *y*. *P*{*Y*=*y*} is the probability that *i*’s influence reaches *j* along the path *y*, which is estimated by the depth-associated attenuation coefficient at the depth from *i* to *j* along the path *y*. The depth-associated attenuation is actually a compounding factor that incorporates the decreasing reaching probability, trustworthiness decay and information corruption. As indicated in the *three-degrees-of-influence* phenomenon (Christakis and Fowler 2007), the influence ceases to have a noticeable effect on people beyond three degrees of separation. Therefore, we set the depth limit *d*_*max*_ to 3 for each influencer. In other words, the influence of any influencer can propagate up to a social horizon of three hops.

Influence also dissipates over time. For example, right after we purchase a new product, we usually feel the excitement and urge to tell our friends (potentially with stronger attempt to influence them). Our enthusiasm and influence potential fade away as time elapses. We model the temporal influence decay as a *time-associated attenuation coefficient* *β*(*t*) as an exponential function of time

5 $$ \beta(t)=e^{-\lambda t},  $$

where *λ* is a user-specified tunable parameter of decay rate. It can be used to account for different products on various social media. When a node is newly activated, a timer is attached to this new influencer and the time is set to 0 at that moment so that we can track the number of hops/steps its influence propagates in its neighborhood. It is noted that the timing for that node acting as a messenger of other influencers has no change.

The next is to model the individual diffusion dynamics. Many real-world networks are thought to be scale-free, in the sense that the degree distribution follows a power law. Therefore, at the network level, it is often seen that some people have much more friends and are much more active in WOM messaging than others. At the individual level, people usually communicate much more frequently with their family members and close friends than casual acquaintances. The contact frequency among friends is strongly correlated with the relationship strength. As a result, WOM messaging from one person to each of her friends generally takes place in an asynchronous manner. To capture the individual temporal diffusion dynamics, we model the heterogeneity of WOM messaging as a *Poisson process*. For a directed edge (*u*→*v*), let *X*_*uv*_ denote the number of times node *u* makes contact with node *v* during one time step. We assume that *X*_*uv*_ follows a Poisson distribution with a rate of *μ*_*uv*_. Its probability mass function can be written as

6 $$ p_{uv}(x)=\left\{ \begin{array}{ll} \frac{\mu_{uv}^{x} e^{-\mu_{uv}}}{x!}, & x=0,1,2\ldots\\ 0, & \text{elsewhere.} \end{array}\right.  $$

Statistically, *μ*_*uv*_ is the average number of times *u* makes contact with *v* per time step. It can be estimated using *out-link weights* of each node to differentiate their activeness and vertex-pair diffusion dynamics. In practice, the weights on edges may have different meanings and properties, and their values may vary significantly. We use 0-1 scaling to bring them into alignment. Specifically, *μ*_*uv*_ is computed as

7 $$ \mu_{uv}=\hat{\mu}+\mu_{u}+\tilde{w}_{uv},  $$

where $\hat {\mu }$ is a tunable parameter that measures the overall activeness of the network, and *μ*_*u*_ and $\tilde {w}_{uv}$ can be interpreted as the *global activeness* of node *u* and *local activeness* of *u*→*v*, respectively. A sensible range for $\hat {\mu }$ is (0,2]. The larger $\hat {\mu }$, the more active the network is. When $\hat {\mu }=2$, the probability that a node contacts with its out-link neighbor at least once in one time step is greater than or equal to 0.865. When $\hat {\mu }$ is too small, the network would be too inactive to facilitate viral marketing. It can be learned using data-driven approaches in practice. We set it to 1 by default. Let *w*_*u*_ denote the sum of all out-link weights of node *u*, and define *w*_*max*_= max*i*∈*Vw*_*i*_ and *w*_*min*_= min*i*∈*Vw*_*i*_. Let *w*_*u*:*max*_ and *w*_*u*:*min*_ denote the maximum and minimum weight among node *u*’s out-links, respectively. Then *μ*_*u*_ and $\tilde {w}_{uv}$ can be calculated as follows

8 $$ \mu_{u}=\frac{w_{u}-w_{min}}{w_{max}-w_{min}},  $$

9 $$ \tilde{w}_{uv}=\frac{w_{uv}-w_{u:min}}{w_{u:max}-w_{u:min}}.  $$

These normalization schemes enable us to differentiate a node’s activeness at both the network and the individual levels. Specifically, for a scale-free network, there exist a few hubs, which are nodes with a degree that greatly exceeds the average. These high-degree nodes are usually more active and more influential, and play an important role in WOM marketing. The global activeness *μ*_*u*_ has a desirable interpretation of the degree distribution. Hubs in a scale-free network would have a significantly larger *μ*_*u*_ than other nodes. On the other hand, if the network of interest is not scale-free, *μ*_*u*_ would be more randomly or normally distributed. Degree-based seeding strategy would be no longer effective. In case *w*_*max*_=*w*_*min*_ (which rarely occurs), we set *μ*_*u*_ to 0.5 for all *u*∈*V*. Similarly, $\tilde {w}_{uv}$ captures node *u*’s local activeness, which is proportional to the weight on the link from *u* to its respective neighbor. If *w*_*u*:*max*_=*w*_*u*:*min*_ (e.g., unweighted networks), we set $\tilde {w}_{uv}$ to 0.5.

Once the rate *μ*_*uv*_ is determined, it is straightforward to calculate the probability with which the WOM message propagates from node *u* to its out-neighbor *v* at one time step as

10 $$ p_{uv}=1-P(X=0)=1-e^{-\mu_{uv}}.  $$

If the propagation is not realized at time step *t*_*i*_, then the probability for the message to be transmitted at time step *t*_*i*+1_ is unchanged due to the memoryless property of Poisson distribution. If *u* has delayed passing on the message to *v* for *delay*_*max*_ time steps (*maximum delay*), it is assumed that *u* has no intention to pass the message on to *v* at all. This is a sensible mechanism reflecting the fact that not everyone is actively engaged in WOM messaging with each of its neighbors at any time step. In addition, we set *delay*_*max*_ to 3 time steps in alignment with the depth limit *d*_*max*_ of 3, which implies that the message would have no noticeable influence even if it is finally propagated after it has been held for 3 or more time steps.

Now we can quantify the influence along a diffusion path at a specific time step. Let $\sigma _{i:x\rightarrow y}^{t,d}$ denote the amount of influence (originated from an influencer *i*) that node *x* passes on to node *y* at time step *t* and depth *d*. Then following a diffusion path from influencer *i*→*j*→*k*→*l*, the influence that nodes *j*, *k* and *l* acquire from node *i* is respectively calculated as

11 $$\begin{array}{@{}rcl@{}} \sigma_{i:i\rightarrow j}^{t_{1},1}&=& e^{-\lambda t_{1}}\times \frac{1}{1^{2}}\times \hat{w}_{ij}, \end{array} $$

12 $$\begin{array}{@{}rcl@{}} \sigma_{i:j\rightarrow k}^{t_{2},2}&=& e^{-\lambda t_{2}}\times \frac{1}{2^{2}}\times \hat{w}_{ij}\times \hat{w}_{jk}, \end{array} $$

13 $$\begin{array}{@{}rcl@{}} \sigma_{i:k\rightarrow l}^{t_{3},3}&=& e^{-\lambda t_{3}}\times \frac{1}{3^{2}}\times \hat{w}_{ij}\times \hat{w}_{jk}\times \hat{w}_{kl}. \end{array} $$

In each equation above, the first term on the right hand side is the temporal influence decay, the second term is the depth-associated influence attenuation, and the rest is the chain product of the normalized influence weights on the corresponding links that constitute the diffusion path from the influencer to the node of interest.

The diffusion process starts with an initial set of influencers (seed nodes) *S*_0_ with |*S*_0_|=*K*, and unfolds in discrete time steps. At each time step, the influence is propagated one hop from a node *u* (parent node) to each out-neighbor *v* (child node) with a probability *p*_*uv*_. Each seed node is assigned an influence value of 1. Each inactive node *v* is initialized with an influence value of 0, and selects an activation threshold *θ*_*v*_ uniformly at random in the range [ 0,1]. Let *A* denote the set of *v*’s in-neighbors who pass on influence to *v*, and let *f*_*v*_(*A*) denote the threshold function of *v*, that is, the total influence that *v* receives from nodes in *A*. Formally, we define *f*_*v*_(*A*) as

14 $$ f_{v}(A)=\min \left(1, \sum\limits_{u\in A} b_{uv}\right), \quad A\subseteq N_{v}^{in},  $$

where *b*_*uv*_ represents the total amount of influence that *v* receives from *u*, and $N_{v}^{in}$ denotes the set of in-neighbors of *v*. Whenever *f*_*v*_(*A*)≥*θ*_*v*_, *v* is activated and turns into an influencer with an influence value of 1. Then it not only continues passing other influencers’ influence as a messenger, but also starts to spread out its own influence as an influencer. The diffusion process stops when the number of hops of influence diffusion of each influencer (including the seed nodes and all activated nodes) reaches the depth limit (*d*_*max*_=3 by default) and no new activation is possible.

### Influence Maximization under MAT Model

The influence-maximization (IM) problem under our MAT model can be described in a general framework as follows: Given a social network *G*=(*V*,*E*,*W*), a budget *K* denoting the seed-set size, and the MAT model on *G* associated with parameters *λ* (temporal decay rate), *d*_*max*_ (depth limit), *p*_*uv*_ for each (*u*→*v*)∈*E* (individual diffusion probability) and *delay*_*max*_ (maximum delay) as input, find a seed set *S*_0_⊆*V* with |*S*_0_|≤*K* such that the expected influence spread *σ*(*S*_0_) is maximized under the MAT model. For viral marketing, the seed nodes are the initial adopters of a new product, and the influence spread is measured by the total number of people in the network who adopt the product in the end of the diffusion process.

The IM problem is NP-hard under the MAT model, which can be proved by reduction. If we set *λ*=0, *d*_*max*_=1, *delay*_*max*_=0, and *p*_*uv*_=1 if *u* is active and *p*_*uv*_=0 if *u* is inactive for any *u*∈*V*, the MAT model then reduces to the classic LT model. Therefore, the IM problem over this class of instances is equivalent to the IM problem under the classic LT model, which is known to be NP-hard (Kempe et al. 2003). This concludes the proof.

It is easy to show that the influence spread function *σ*(·) for the MAT model is monotone. For each run of the diffusion process, the threshold *θ*_*v*_ (for each *v*∈*V*) is randomly selected and fixed. When the seed set *S*_0_ grows, the final active set *ϕ*(*S*_0_) also grows under these fixed thresholds, due to the monotonicity of *f*_*v*_(*S*). Finally, *σ*(*S*_0_) is simply the average of the size of all final active sets among all possible threshold values selected in different runs, and thus is monotone.

However, it is hard to prove that *σ*(·) for the MAT model is also submodular. The widely-used approach is to construct a *live-edge graph model* (Chen et al. 2013) that is equivalent to the diffusion model of interest. In a live-edge graph, any active node should be reachable from at least one seed node along at least one *live-edge path* consisting entirely of active nodes chained up from one to another. It implies that any active node (except seed nodes) should have at least one active neighbor, which makes it infeasible to apply this approach to our MAT model since a node without any active neighbors may still be activated by indirect influence in the MAT model. For now, we conjecture that *the influence spread function σ(·) for our MAT model is submodular*. We leave the proof as future work.

To address the IM problem, we need to find the “*optimal*” seed set. Let us first clarify two important concepts: *aggregation* and *overlap*. Aggregation means that an inactive node is reinforced with more WOM exposures and receives more influence. When it accumulates enough influence (greater than or equal to its threshold), it becomes active and starts to spread its own influence as an influencer. Overlap means that an influencer spreads its influence to any nodes that have been activated by other influencers. On one hand, if seed nodes are closely clustered, they may potentially have large overlaps, which leads to deterioration in performance. On the other hand, if seed nodes are totally isolated, they can not achieve aggregation effect, which results in a detriment to new activations as well. Therefore, the ideal selection of the *K* seed nodes would be the *K* most influential nodes such that: (1) each of them achieves *individual* influence spread as large as possible; (2) they should be far enough away from each other to minimize potential overlaps; and (3) they should be close enough to reinforce aggregation effects. Using these guidelines in our seeding strategy, we develop a novel algorithm, *IV-Greedy*, to tackle the IM problem under the MAT model.

### IV-Greedy algorithm

As discussed in the previous section, MC-Greedy (Kempe et al. 2003) is a simple greedy hill-climbing algorithm, using Monte Carlo (MC) simulation to estimate the influence spread. Although its computational cost is extremely high due to the overhead of MC simulation, it achieves the largest influence spread under the IC and LT models among all state-of-the-art algorithms (Chen et al. 2013). Regardless of its low efficiency, the *greedy* strategy used in MC-Greedy makes sense and is highly effective. We develop IV-Greedy borrowing the greedy strategy of MC-greedy but replacing MC simulation by using the *influence vector* of each node.

The influence vector of a node captures where and how much influence that node spreads over its neighborhood based on a *nonstochastic* version of the MAT model, in which we do not consider individual diffusion dynamics (i.e., let *p*_*uv*_=1 for any *u*∈*V*), or the activation of the nodes (i.e., only *i* is an influencer; all other nodes remain messengers). In addition, we ignore the temporal diffusion decay (i.e., let *λ*=0) when generating the influence vectors such that our IV-Greedy is more robust with different decay rates. We develop a modified depth-limited search algorithm, called *IV-Builder*, to explore the diffusion tree of each node individually, and generate an influence vector for each node, which records its influence spread to all the nodes in its neighborhood within a depth limit of 3 (by default). IV-builder uses the same approach as we use to generate the influence matrix for community detection in (Wang and Street 2014; 2015a). The only difference exists in the weight normalization schemes. For community detection, we normalize each in-weight of a node by the *maximum* in-weight of that node to capture the *relative susceptibility* of the node to its in-neighbors, and the influence vectors are used to differentiate vertex-pair similarity. For viral marketing, we normalize each in-weight of a node by the *total* in-weight of that node to quantify the *absolute influence* transmitted from each in-neighbor, and the influence vectors are used to find out which nodes are more likely to be activated. Due to space limit, we refer the interested reader to (Wang and Street 2015a) for implementation details on influence-vector generation.

We use these influence vectors as a proxy for the *stochastic* influence-diffusion process to avoid the expensive MC simulation. Specifically, We define the *influence score* of a node as the sum of all elements in its influence vector, which represents an estimate of its total influence spread. This influence score can be used to differentiate the influence significance of the nodes in the network. An intuitive solution is to select the top-*K* nodes of the highest influence scores as the seeds. Unfortunately, it does not work well. Like those high-degree nodes, the nodes of high influence scores may be clustered or too close to each other, leading to large overlaps of influence spread. We need to somehow separate them from each other to minimize overlaps. The strategy that we use in IV-Greedy is a greedy strategy similar to MC-Greedy, in which we sweep over the influence vector of each node to repeatedly pick the node with the *maximum marginal gain* and add it to the seed set until all *K* seeds are found. The pseudocode is shown in Algorithm 2. 
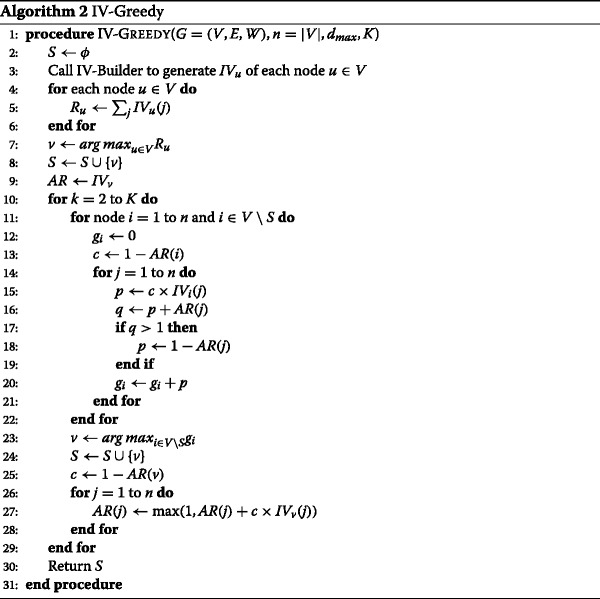


In IV-Greedy, each node is indicated by its node index from 1 to *n*. IV-Greedy starts with an empty seed set (Line 2), and then generate the influence vector *IV*_*u*_ for each node *u*∈*V* (Line 3). *IV*_*u*_ is an *n*-element array, in which element *IV*_*u*_(*j*) represents the amount of influence node *u* exerts on node *j*. For each node *u*, we get its influence score *R*_*u*_ by summing up all elements in its influence vector *IV*_*u*_ (Lines 4-6). We find the node *v* that has the highest influence score (Line 7), and add it to the seed set *S* as the first seed (Line 8). Then we make a copy of *IV*_*v*_ to an array *AR*, which is used as a representation of the *collective* influence distribution of currently selected seeds. To find the next seed, we sweep over each node *i*∈*V*∖*S* and compute the marginal gain *g*_*i*_ of adding *i* to *S* individually. We compute its *benefit factor* *c*=1−*AR*(*i*) (Line 13). Recall that *AR*(*i*) represents the amount of influence *i* receives so far, which can be also regarded as the probability activating *i*. For example, if *AR*(*i*)=1, it means that *i* has been activated, then selecting *i* as a seed has no benefit to the overall influence spread. Then for each element *IV*_*i*_(*j*), we get *p*=*c*×*IV*_*i*_(*j*) (Line 15), which represents the marginal gain that node *j* gets. However, remember that the threshold function *f*_*v*_(*S*) is defined within the range [ 0,1]. If the amount of influence that node *v* receives is greater than 1, it is set to 1 since *v* has been activated and more influence on *v* is of no more effect or benefit. Therefore, we add *p* to *AR*(*j*) to get *q* (Line 16), which represents the accumulated influence that node *j* receives. If *q* is greater than 1, we set the actual marginal gain *p*=1−*AR*(*j*) (Lines 17-19). Then we add *p* to *g*_*i*_, which represents the total marginal gain of adding node *i* to *S*. Once we get the marginal gain of each node *i*∈*V*∖*S* (Lines 11-22), we select the node that produces the maximum marginal gain as a seed and add it to *S* (Lines 23-24). Then we update *AR* by calculating the benefit factor *c* and adding the marginal gain to each element in *AR* (Lines 25-28). We repeat the process described above (*K*−1) times to find the (*K*−1) seeds, which along with the first seed node constitute a full seed set *S*.

## Experiments

We conduct experiments on four widely-used real-life network datasets to evaluate our MAT model and the performance of IV-Greedy, and compare it against a set of baseline algorithms on both influence spread and time efficiency. The code is written in Visual Basic and all experiments are carried out on a regular desktop PC with Intel(R) Core(TM) i5-4670 CPU @ 3.40 GHz and 8.0 GB memory under Windows 7 64-bit OS.

### Network description

To evaluate the applicability of our model and algorithms, we employ four real-life networks with different combinations of link directionality and weights. We list their statistics in Table 1. *PGP* (Boguna et al. 2004) is an undirected/unweighted network of users of the Pretty-Good-Privacy algorithm for secure information interchange. Each node represents a user, and each edge connects a pair of users of interest who have assigned public keys of another based on trust between them. It is a single connected component with relative clear community structure. *NetHEPT* (HEPT Collaboration Network 2009) is an undirected/weighted collaboration network from the paper lists extracted from “High Energy Physics (Theory)” section of the e-print arXiv from 1991 to 2003. Each node represents a unique author, and each edge represents co-authorship of the two authors of interest, weighted by the number of papers they have co-authored. This network has been frequently used in previous work (Chen et al. 2010; Goyal et al. 2011b). *WikiVote* (Wikipedia Who-vote-whom Network 2010; Leskovec et al. 2010) is a directed/unweighted who-vote-whom network from Wikipedia. Nodes in the network represent Wikipedia users and a directed edge from nodes *i* to *j* represents that user *i* voted on user *j*. This link direction does not reflect the direction of influence flow. User *i* voting on user *j* is actually because *j* has influence on *i*, as analogous to that of citing paper *i* and cited paper *j*. Therefore, we reverse the link direction to reflect the actual influence flow. This network has a giant component and a set of 23 small ones. The last network dataset is *C.elegans* (White et al. 1986). It is a directed/weighted neural network of the nematode worm C.elegans.

**Table 1:** Statistics of network datasets

Dataset	PGP	NetHEPT	WikiVote	C.elegans
Directed	No	No	Yes	Yes
Weighted	No	Yes	No	Yes
Nodes	10,680	15,233	7,115	453
Directed links	0	0	97,835	2,025
Undirected edges	24,316	31,376	2,927	0
Average out-degree	4.6	4.1	14.6	4.5
Maximum out-degree	205	64	457	145
Average weight	1	1.9	1	2.3
Maximum weight	1	119	1	114
Connected components	1	1,781	24	1
Average component size	10,680	8.6	296.5	453
Largest component size	10,680	6,794	7,066	453

### Baseline heuristics

The simplest and naive baseline is to select nodes uniformly at random (hereafter referred to as RANDOM). Not surprisingly, this algorithm is very unstable and does not perform well in terms of influence spread. The most frequently used is the *degree-centrality* heuristic (hereafter referred to as DEGREE), in which the seed nodes *v* are chosen in descending order of out-degrees $d_{v}^{out}$. This seeding strategy is simple but effective. Empirical studies (Kempe et al. 2003; Hinz et al. 2011) show that DEGREE results in larger influence spread than other centrality-based heuristics, such as distance centrality and betweenness centrality. A common feature of these centrality-based algorithms is that they rely solely on one specific structural property of the network without considering the diffusion dynamics. In fact, many of the highest-degree nodes may be clustered and have potentially large overlaps of influence spread, which leads to deterioration in performance.

The next baseline is the Top-*K* algorithm. We present its pseudocode in Algorithm 3. It sweeps over each node *u*∈*V* to compute the influence spread of each node individually (Lines 2-9), using Monte Carlo simulation (Lines 4-7). Then, it selects the top *K* nodes with the largest *individual* influence spread (Lines 10-13). It is worth noting that the top-*K* nodes that produce the largest influence spread *individually* is usually not the same as the *K* seed nodes that produce the largest influence spread *together*. For example, if two top influencers are so close to each other that their influence spreads have a large overlap, it is not a good idea to select both of them as seed nodes. While the DEGREE algorithm relies solely on the structural properties of the network without considering the diffusion dynamics, the Top-*K* algorithm relies solely on the diffusion dynamics without considering the network structure.


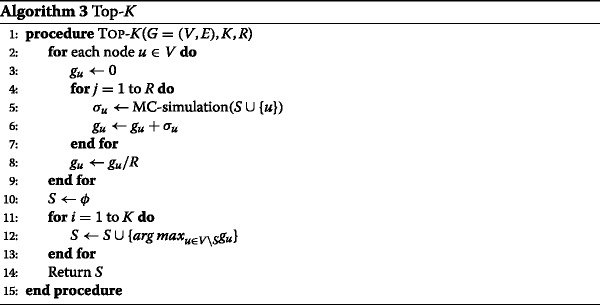


MC-Greedy (as shown in Algorithm 1) is the last baseline heuristic. It considers both the diffusion dynamics and the network structure (implicitly). One may notice that the Top-*K* algorithm is actually the first round of the *K* rounds in MC-Greedy except that MC-Greedy selects only top one influencer instead of top *K* influencers. It continues exhaustively running MC simulation to find the next seed node that produces the *maximum marginal gain* in influence spread. This step iterates until a set of *K* seed nodes is found. MC-Greedy usually achieves the largest influence spread of the *K* seed nodes at the collective level. However, it suffers from a very high computational cost, which makes it infeasible even for medium-sized networks.

### Performance comparison

We run experiments on the four network datasets to compare the performance of IV-Greedy against the four baseline algorithms in terms of influence spread and time efficiency. As discussed in the previous section, we set both *d*_*max*_ and *delay*_*max*_ to 3, and treat them as built-in parameters of the MAT model. We also set the network activeness parameter $\hat {\mu }$ to 1 as defaulted. The temporal decay rate *λ* is regarded as a user-specified parameter. The larger *λ*, the faster temporal decay of influence. We set it to 0.2 for performance comparison of different algorithms, and then evaluate its effect on influence spread with different values. In addition, both MC-Greedy and Top-*K* need to run MC simulation *R*_1_ times in each round of influence-spread estimation. Once an algorithm produces the seed set it finds, we run MC simulation *R*_2_ times to estimate the influence spread of each algorithm for comparison. In our experiments, we set both *R*_1_ and *R*_2_ to 1000. In particular, due to the extremely low efficiency of MC-Greedy, we only report its results for C.elegans dataset.

#### Influence spread

**Fig. 1:**
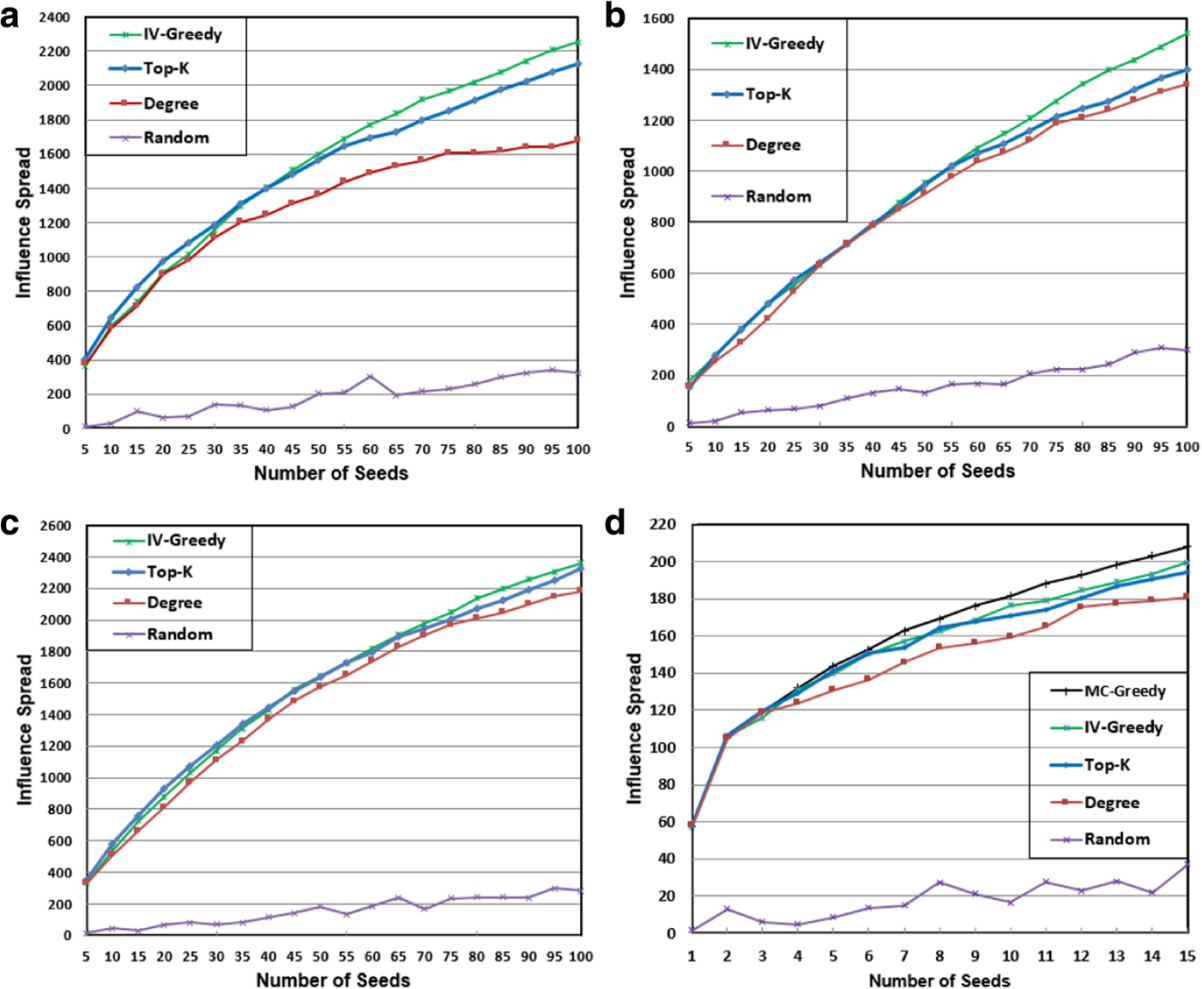
Performance comparison on influence spread. **a** PGP dataset, **b** NetHEPT dataset, **c** WikiVote dataset, and **d** C.elegans dataset

We illustrate in Fig. 1 the experimental results of the four network datasets. Each curve shows the variation of the influence spread with respect to the seed-set size *K*. *K* is a user-specified parameter. In practice, *K* is closely related to the marketing budget. For diffusion research, we choose *K* primarily based on the network size. Specifically, we vary the number of seeds *K* from 5 to 100 in an increment of 5 for PGP, NetHEPT and WikiVote since they have 10680, 15233, and 7115 nodes, respectively. *K* is varied from 1 to 15 in an increment of 1 for C.elegans since it has only 453 nodes.

It is not surprising that RANDOM has the worst performance on all datasets. The degree-centrality heuristic, DEGREE, greatly improves the influence spread, which is 4-8 times better than RANDOM. It makes sense that the well-connected nodes (social hubs) facilitate influence diffusion with their high reach to others. Top-*K* is analogous to DEGREE in the sense of targeting the group of most influential nodes who achieve largest influence spread *individually*. It achieves an improvement of 28.4*%*,3.8*%*,6.5*%*, and 7.3*%* over DEGREE on PGP, NetHEPT, WikiVote, and C.elegans, respectively. However, its improvement on influence spread comes with a huge sacrifice on efficiency since it relies on expensive MC simulation. Both DEGREE and Top-*K* fail to capture the fact that many of the highest-degree or most influential nodes may be clustered, which results in large overlaps of influence spread. IV-Greedy incorporates the mechanism to minimize overlaps, and achieves the best performance in the comparison with RANDOM, DEGREE, and Top-*K*. The larger seed-set size, the greater improvement. Specifically, for *K*=100 for PGP/NetHEPT/WikiVote and *K*=15 for C.elegans, IV-Greedy beats DEGREE by 35.1*%*,14.9*%*,8.6*%* and 10.8*%*, and outperforms Top-*K* by 5.3*%*,10.7*%*,1.6*%* and 3.3*%*, respectively. It is only inferior to MC-Greedy by 3% on C.elegans. As shown in the comparison of running time, IV-Greedy runs several orders of magnitude faster than MC-Greedy (and Top-*K*) on the other hand.

Moreover, we list in Table 2 the 95% confidence intervals (CI) for the expected influence spread of the four network datasets, based on 1000 runs of MC simulation for each dataset. We also perform *t*-test at the 0.05 significance level on IV-Greedy versus DEGREE and IV-Greedy versus Top-*K*, respectively. The corresponding *p*-values for each dataset are calculated and included in Table 2 as well. Clearly, both the confidence intervals and the *p*-values indicate that the improvement of IV-Greedy over DEGREE and Top-*K* is statistically significant for all datasets.

**Table 2:** Ninety-five percent confidence intervals and *p*-values for the expected influence spread of the four network datasets based on 1000 runs of MC simulation for each dataset

Dataset	Measure	Algorithm		
		DEGREE	Top-*K*	IV-Greedy
PGP	95% CI	1666.3 ± 6.2	2139.3 ± 6.7	2251.7 ± 7.0
	*p*-value	0	3.4e-103	
NetHEPT	95% CI	1339.8 ± 5.7	1390.6 ± 5.4	1539.7 ± 6.4
	*p*-value	0	5.0e-207	
WikiVote	95% CI	2175.4 ± 6.6	2323.6 ± 6.6	2361.6 ± 6.7
	*p*-value	1.5e-246	3.4e-15	
C.elegans	95% CI	180.7 ± 0.7	193.9 ± 0.8	200.3 ± 0.8
	*p*-value	1.4e-215	2.3e-26	

#### Running time

An analysis of the time complexity of the proposed algorithms is given as follows. Let *n* and *m* denote the number of nodes and edges in the network, respectively. For DEGREE, it is straightforward to sweep over each node K times to select the top *K* nodes of the highest degrees as the seed set. Therefore, the time complexity of DEGREE is *O*(*Kn*). IV-Greedy (as shown in Algorithm 2) needs to call IV-builder to generate an influence vector for each node first. Let *L* denote the average length of influence vectors. *L* is determined by the average node out-degree *b*, depth limit *d*_*max*_ and and the community structure. As elaborated in our previous work (Wang and Street 2014; 2015a), IV-builder may take *O*(*b*^*d*^) time exploring the diffusion tree of each node. In most cases, the time complexity of IV-builder is *O*(*Ln*), and *L* is way smaller than *n*, especially for large-scale networks. Therefore, we usually do not maintain each influence vector in an *n*-element array in practice, but only keep the non-zero influence values in a compact dynamic array instead. Then in Line 11 of IV-Greedy, the number of iterations is reduced to *L* from *n*. Therefore, the time complexity of IV-Greedy is *O*(*KLn*).

Both Top-*K* and MC-Greedy reply on MC simulation. Each run of MC simulation takes *O*(*m*) time. Top-*K* (as shown in Algorithm 3) needs to sweep over each node to compute the influence spread individually by running MC simulation R times for each node, and thus it has a time complexity of *O*(*nRm*). As we can see, Top-*K* already has a very high computational cost. Unfortunately, MC-Greedy (as shown in Algorithm 1) is much more time consuming than Top-*K*. Top-*K* is just the first round in MC-Greedy finding the first seed. This process has to repeat *K* rounds to find the *K*-node seed set. In each round, the node that generates the maximum marginal gain is selected and added to the seed set. Therefore, the time complexity of MC-Greedy is *O*(*KnRm*). In fact, in the *k*^*th*^ round of repetition, the seed-set size increases to *k*. The time that each run of MC simulation in the *k*^*th*^ round takes is more than *k* times of that in the first round. In other words, the actual running time of MC-Greedy could be several *K*^2^ times of that of Top-*K*, which makes it infeasible even for medium-sized networks of thousands of nodes.

**Fig. 2:**
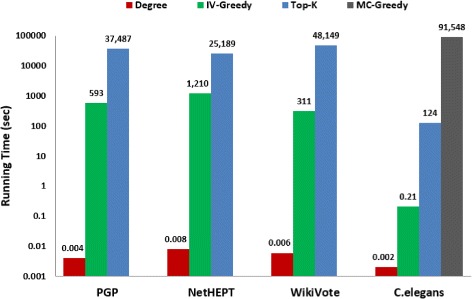
Performance comparison on running time (CPU seconds)

In Fig. 2, we illustrate the running time of the algorithms on the four network datasets. A seed-set size of *K*=100 is set for the PGP, NetHEPT, and WikiVote datasets. *K* is set to 15 for the C.elegans dataset. It is noted that the *Y*-axis is in logarithmic scale. Clearly, DEGREE is the fastest algorithm. It finishes almost instantly on all datasets. IV-Greedy significantly outperforms Top-*K*. Specifically, IV-Greedy is 63, 21, 155 and 590 times faster than Top-*K* on PGP, NetHEPT, WikiVote and C.elegans, respectively. Not surprisingly, MC-Greedy is the slowest one. As we can see on the C.elegans dataset of only 453 nodes and 2025 edges, while IV-Greedy takes only 0.2 seconds to find a set of 15 seed nodes, Top-*K* takes 2 minutes 4 seconds, but MC-Greedy takes more than 25 hours. Due to such a high computational cost, we are not able to run it on the other three datasets. Overall, IV-Greedy is the best performing algorithm in terms of both influence spread and running time.

### Adoption rate

**Fig. 3:**
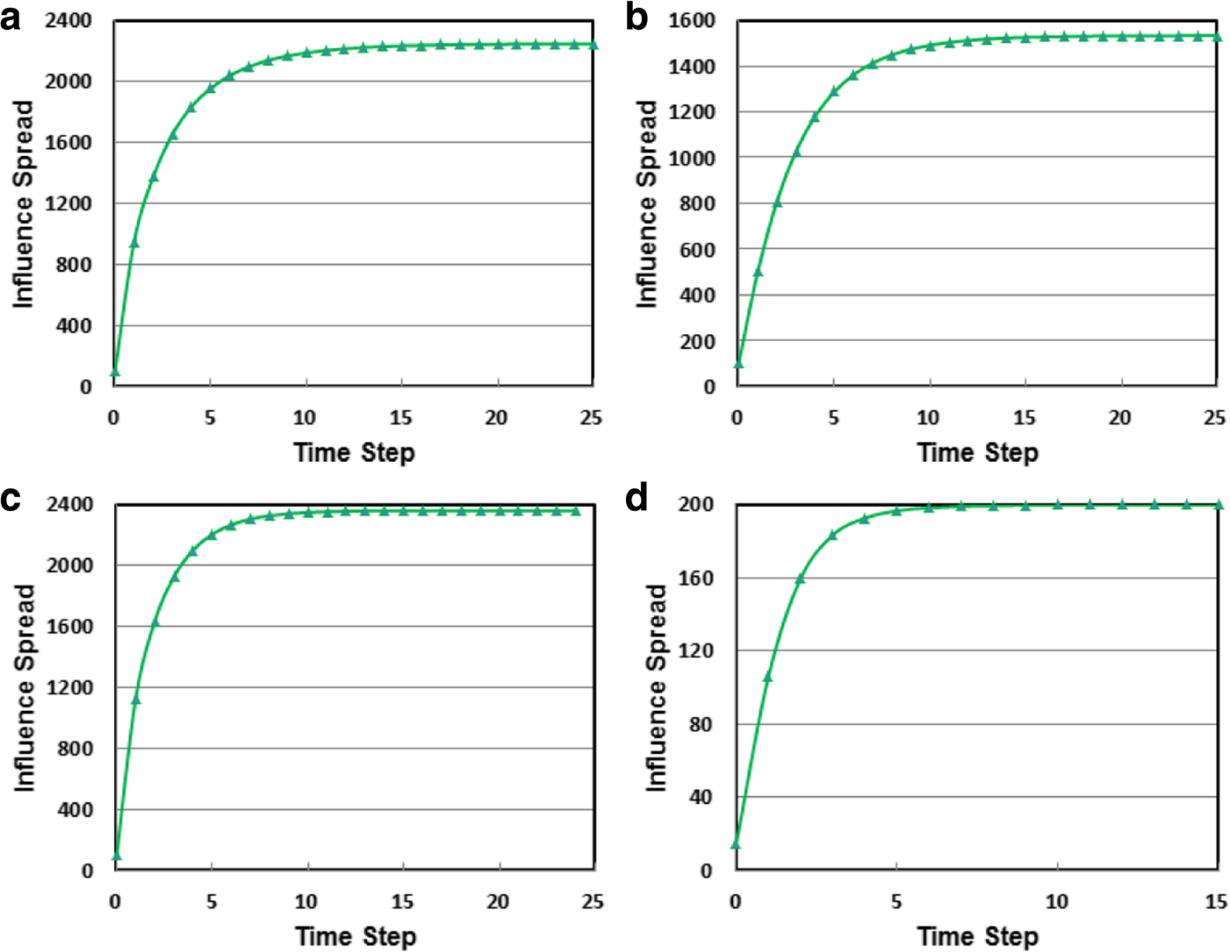
Adoption rate achieved by IV-Greedy. **a** PGP dataset, **b** NetHEPT dataset, **c** WikiVote dataset, and **d** C.elegans dataset

Marketers are concerned about not only the influence spread but also the adoption rate, which is usually measured by the number of adopters in a given time period. How fast the influence spreads is a critical factor for a viral marketing campaign, especially when there exist competing products in the same social network. It is interesting to observe the rate of adoption under our MAT model. We show in Fig. 3 the influence spread over time on the four network datasets with IV-Greedy as the seeding strategy. Again, the seed-set size is set to 100 for PGP, NetHEPT and WikiVote, and 15 for C.elegans.

As we can see, the adoption rate shows similar pattern on all datasets. The influence diffuses at a high-speed rate at early stage, and the speed decreases monotonically. The influence spread reaches the saturation level in about 10 to 15 time steps. At *t*=5, the influence spread arrives at 87%, 84%, 93.4*%*, and 98.3*%* of the maximum on PGP, NetHEPT, WikiVote, and C.elegans, respectively. At *t*=10, this percentage increases to 97.4*%*, 97.3*%*, 99.5*%*, and 100%, respectively. It is observed that WikiVote and C.elegans achieve higher adoption rates than PGP and NetHEPT, which can be roughly explained by the high cohesiveness of WikiVote and the small network size of C.elegans. This follows our intuition. In practice, marketers need to carefully determine the period of a time step. It could be a day, a week, or a month, etc. The influence decay rate and individual contact frequency may vary accordingly.

### Parameter analysis and model comparison

**Fig. 4:**
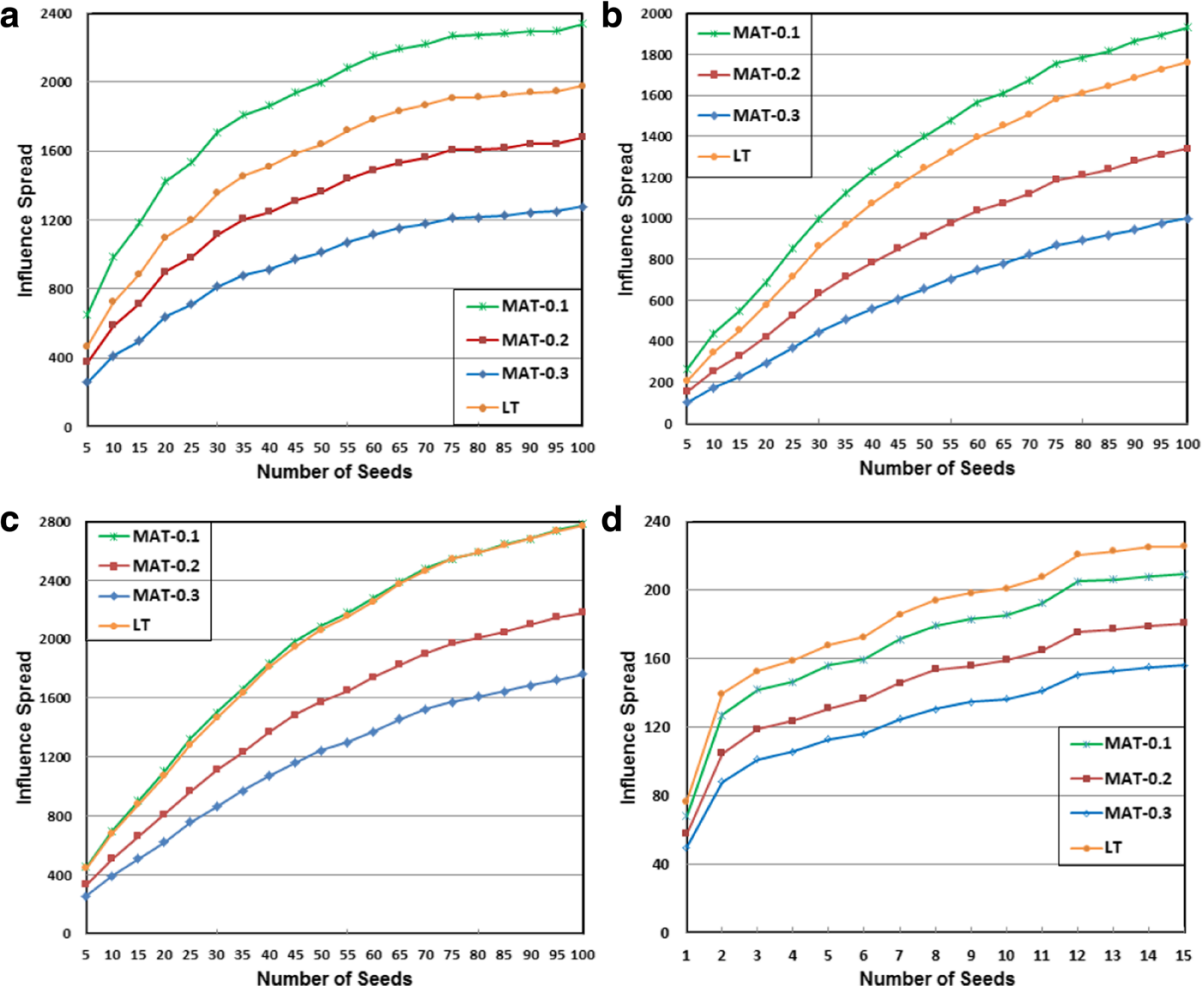
Influence spread achieved by DEGREE under the classic LT model and the MAT model with different temporal influence decay rates. **a** PGP dataset, **b** NetHEPT dataset, **c** WikiVote dataset, and **d** C.elegans dataset

The temporal decay rate *λ* is a user-specified parameter in our MAT model. The value of *λ* can be varied to account for different products on different social networks. For a particular application, it is possible to use a data-driven approach to learn the appropriate value of *λ*. We evaluate the impact of *λ* on influence spread using different decay rates under the MAT model. We also include the classic LT model (Kempe et al. 2003) as a baseline for model comparison. DEGREE is a fairly effective and generic heuristic that is applicable to both the MAT model and the LT model. Moreover, it always produces the same seed set, which enables us to perform a fair comparison on different models. Therefore, we use DEGREE in this experiment. We set *λ* to 0.1, 0.2, and 0.3, respectively. The experimental results are illustrated in Fig. 4.

As expected, *λ* in our MAT model has significant impact on influence spread. A larger *λ* indicates faster temporal decay of influence, and thus results in smaller influence spread. It is good to see that *λ* in the MAT model enables us to gauge the influence spread in a reasonably large range on all datasets. When *λ* increase from 0.1 to 0.3, influence spread drops 45.4*%*, 48.1*%*, 36.7*%* and 25.4*%* on PGP, NetHEPT, WikiVote and C.elegans, respectively.

Bhagat et al. (2012) evaluate the influence spread of several models. Their findings suggest that the classic LT model overestimates the influence spread by large amounts. As we can see, when *λ*=0.2, the influence spread under the MAT model is already 15%, 24%, 21.3*%* and 19.9*%* smaller than the LT model on PGP, NetHEPT, WikiVote and C.elegans, respectively. When *λ*=0.3, the influence spread under the MAT model drops to 35.4*%*, 43.2*%*, 36.4*%* and 31% smaller than the LT model on PGP, NetHEPT, WikiVote and C.elegans, respectively. We believe that our MAT model is more powerful than the LT model on prediction of influence spread. It is our future work to develop an effective data-driven approach to learn *λ* from training data and then use it in the MAT model to predict the diffusion.

## Conclusion

In this paper, we propose a novel *multiple-path asynchronous threshold* (MAT) model for viral marketing in social networks. It differs from existing diffusion models in several aspects. We quantitatively measure influence and keep track of its spread and aggregation during the diffusion process. Our MAT model captures both direct and indirect influence, depth-associated influence attenuation, temporal influence decay, and individual diffusion dynamics. Our work is an important step toward a more realistic diffusion model. Further, we develop an effective and efficient heuristic, IV-Greedy, to tackle the influence-maximization problem. Our experiments on four real-life networks demonstrate its excellent performance in terms of both influence spread and time efficiency.

Our work provides preliminary but significant insights and implications for marketing practice. Firs of all, unlike other diffusion models relying exclusively on the direct influence from influencers, our model draw managerial attention to messengers who play an important role in spreading indirect influence in viral marketing. Our model offers an algorithmic view of the complex WOM communications, as described in the *network coproduction model* (Kozinets et al. 2010). Second, our work provides pragmatic implications for how marketers should plan and leverage WOM campaigns. They need to take into consideration depth-associated influence attenuation and temporal influence decay when determining the time period/steps. Specifically, it is necessary for the marketers to find out the decay rate attuned to the product and/or social media of interest. Third, to fully grasp the effect of the WOM marketing, marketers need to look beyond the network structure and incorporate the weight information on edges to capture the network activeness and individual diffusion dynamics. Finally, our work suggests that marketers can create an efficient seeding strategy that achieves larger influence spread than the high-degree seeding. They need to consider both individual influence spread and the network structure to minimize the potential overlaps.

Our work opens up several directions for future work on diffusion research. First, the conjecture on submodularity of the influence-spread function under the MAT model needs rigorous proof. Second, scalable heuristics need to be developed for large-scale networks, especially for dense networks and large seed-set size. More importantly, it is necessary to validate the MAT model with more real-life datasets from diverse domains. It is desirable to have the dataset that contains both network connectivity and action traces of a large set of instances. Then we can partition the instances into a training set and a test set, and use the training set to learn model parameters, such as the temporal influence decay rate and the network activeness. Finally, we use those learned parameter values in the MAT model to predict the influence spread for the instances in the test set, and evaluate the prediction accuracy based on the actual influence spread. This would be a practical mechanism to not only validate the model but also build an effective prediction model. A lot of interesting work can follow.
